# Simultaneous photocatalytic and microbial degradation of dye-containing wastewater by a novel g-C_3_N_4_-P_25_/photosynthetic bacteria composite

**DOI:** 10.1371/journal.pone.0172747

**Published:** 2017-03-08

**Authors:** Xinying Zhang, Yan Wu, Gao Xiao, Zhenping Tang, Meiyin Wang, Fuchang Liu, Xuefeng Zhu

**Affiliations:** 1 College of Environment and Resources, Fuzhou University, Fuzhou, Fujian, PR China; 2 Research Institute of Photocatalysis, State Key Laboratory of Photocatalysis on Energy and Environment, Fuzhou University, Fuzhou, China; 3 Section Sanitary Engineering, Department of Water Management, Faculty of Civil Engineering and GeoSciences, Delft University of Technology, Delft, Netherlands; Institute of Materials Science, GERMANY

## Abstract

Azo dyes are very resistant to light-induced fading and biodegradation. Existing advanced oxidative pre-treatment methods based on the generation of non-selective radicals cannot efficiently remove these dyes from wastewater streams, and post-treatment oxidative dye removal is problematic because it may leave many byproducts with unknown toxicity profiles in the outgoing water, or cause expensive complete mineralization. These problems could potentially be overcome by combining photocatalysis and biodegradation. A novel visible-light-responsive hybrid dye removal agent featuring both photocatalysts (g-C_3_N_4_-P_25_) and photosynthetic bacteria encapsulated in calcium alginate beads was prepared by self-assembly. This system achieved a removal efficiency of 94% for the dye reactive brilliant red X-3b and also reduced the COD of synthetic wastewater samples by 84.7%, successfully decolorized synthetic dye-contaminated wastewater and reduced its COD, demonstrating the advantages of combining photocatalysis and biocatalysis for wastewater purification. The composite apparently degrades X-3b by initially converting the dye into aniline and phenol derivatives whose aryl moieties are then attacked by free radicals to form alkyl derivatives, preventing the accumulation of aromatic hydrocarbons that might suppress microbial activity. These alkyl intermediates are finally degraded by the photosynthetic bacteria.

## Introduction

Large amounts of dye-containing wastewaters are generated during processes involved in the manufacture, utilization (particularly by textile, paper, and carpet industries), and disposal of dyes [1]. These wastewaters pose substantial environmental hazards due to their strong colors, high COD (ca. 20% of which is due to dyes and 80% to auxiliary dyeing agents such as sodium acetate and starch) and the complex chemical composition of the discharged effluent [2–4]. Hence, to avoid environmental problems there is a clear need to treat dye-containing wastewater rigorously. Reactive dyes, especially those containing azo-groups, constitute a large proportion of dye contaminants and pose particular problems [1]. Azo dyes have a chromophore consisting of a pair of conjugated aryl moieties linked by an azo (-N = N-) bond, which absorbs certain wavelengths of visible light but reflects others [5]. Because this light absorption depends on the conjugation of the system through the azo group, the destruction of the azo group is an essential step in the decolorization and decontamination of dye-containing wastewater. However, the complex aromatic structures of typical azo dyes make them resistant to light-induced fading and biodegradation [6].

Because of their low costs and energy requirements, biological treatments involving aerobic or anaerobic microorganisms have been widely used to treat wastewaters containing azo dyes. However, these methods are time consuming and incomplete mineralization, toxic and carcinogenic by-products from the cleavage of the azoic bond. Aerobic microbes, including various fungi and algae, have been used to remove azo dyes by adsorption rather than biodegradation, i.e. without destroying the dyes’ molecular structure [7]. Conversely, some anaerobic biological treatments have been shown to destroy the chromophores enzymatically, and decompose a very wide range of organic compounds. However, anaerobic processes may generate arylamine products that can be even more toxic and mutagenic to aquatic living systems than the original azo dyes. Moreover, the products may be resistant to further degradation under anaerobic conditions [8–11].

Recently, advanced oxidation processes (AOPs), especially photocatalytic degradation using various semiconductor oxides, have been developed for removing dyes in contaminated wastewater [12–15]. In a photocatalytic process, a semiconductor is irradiated with photons whose energy is equal to or greater than its band gap, generating electron/hole pairs. Holes (h_1VB_) can react with H_2_O or OH^-^ that adsorb to the semiconductor’s surface, generating hydroxyl radicals, while electrons (e_2CB_) are captured by oxygen or other oxidants in the system, generating superoxide, hydrogen peroxide, hydroxyl and/or other anionic radicals [16]. These free radicals are highly reactive towards diverse contaminants. Among the photocatalysts, TiO_2_ has demonstrated to be the most popular photocatalyst for potential commercialization. However, the large band gap energy of 3.2 eV and the high recombination rate of electron–hole pairs have hindered its wide applications. Hence, various research groups have tried to improve its photocatalytic activity using different approaches such as element doping, coupled semiconductor, and metal deposition[17,18]. Recently, graphite-like carbon nitride with high thermal, biomedical non-toxicity, biocompatible and chemical stability together with a moderate bandgap energy of 2.7–2.8 eV has demonstrated to be effective for solar energy conversion[19]. And it has been combined with various semiconductors including ZnO, CdS, TiO_2_, Fe_3_O_4_, SnO_2_, Cu_2_O and WO_3_, and its use has been explored in supercapacitors, solar cells, gas sensors, batteries and photocatalysts[20].It is expected that coupling TiO_2_ with g-C_3_N_4_ would be able to significantly improve the visible light absorption, carriers' separation, and capability for photooxidation by the created interfaces.

However, photocatalytic systems are indiscriminate; it is difficult to ensure that energy is not wasted by generating a series of products that are excessively oxidized or not readily biodegradable [21, 22]. Partly for this reason, most attempts to develop photocatalytic systems to treat dye-containing wastewater have focused on sequential coupling approaches [23–27,14]. A major complicating factor is that dye-containing wastewater often contains refractory and/or inhibitory organic compounds in a biodegradable matrix. Thus, these approaches cannot overcome all of the limitations of AOP or biotechnology, and provide straightforward, cheap and efficient processes. Another approach that has been tested in several studies (particularly for mineralizing chlorophenol) is to use systems consisting of microorganisms and photocatalysts adsorbed on a sponge. This format system had achieved remarkable results in phenolic wastewater under visible light [21, 28–30].

In principle, coupling photocatalytic and biodegradation systems could be highly advantageous for treating dye-containing wastewater. We therefore tested a novel strategy involving immobilization of graphitic carbon nitride-TiO_2_ (g-C_3_N_4_-P_25_) and photosynthetic bacteria in alginate to form a composite with both photocatalytic and biological oxidation activities. The composite’s structure was evaluated and characterized using N_2_ sorption isotherms, X-ray diffraction (XRD), and scanning electron microscopy (SEM). In addition, to evaluate its ability to treat wastewater contaminated with azo dyes, the composite was incubated with solutions containing reactive brilliant red X-3b and controlled COD levels (established by adding glucose). Finally, the composite’s mechanism of action was explored by monitoring the degradation of the reactive brilliant red X-3b and the rate of COD removal while simultaneously analyzing the intermediates formed during the oxidation process by gas chromatography-mass spectrometry (GC-MS) and UV-visible spectrophotometry.

## Materials and methods

### Chemicals

Reactive brilliant red X-3b (C_19_H_10_Cl_2_N_6_Na_2_O_7_S_2_,λ_max_ = 540nm) was purchased from Future Reagents (Shanghai, China) and used without further treatment. P_25_ was purchased from Aladdin (Shanghai, China). All other chemicals were of analytical grade. The photosynthetic bacteria used were a commercial strain of the genus Rhodospirillum.

### Microorganism and cultivation

The Rhodospirillum was grown in a basal synthetic medium containing (per liter) 3 g sodium acetate, 1 g NaHCO_3_, 1 g NH_4_Cl, 0.5 g Na_2_HPO_4_, 0.2 g MgSO_4_, 1 g NaCl, 0.05 g FeSO_4_·7H_2_O and 0.5 g yeast extract, supplemented with 1 mL/L of a trace nutrient solution containing (per liter): 0.7 g H_3_BO_3_, 0.389 g MnSO_4_·H_2_O, 0.188 g NaMoO_4_·2H_2_O and 0.01 g Cu(NO_3_)_2_ .3H_2_O. The bacteria were cultivated in this medium with illumination by a 100 W fluorescent lamp in a 1000mL conical flask for 3–4 day, then harvested by centrifugation (5000 rpm, 10 min), washed aseptically with deionized water and stored at 4 °C [31].

### Preparation of g-C_3_N_4_-P_25_ hybrid photocatalyst

Sufficient melamine for our experimental purposes (ca. 10 g) was placed in an aluminum crucible with a cover to prevent sublimation of melamine, then heated to 550 °C for 4 h at 2.3 °C/min in a muffle furnace in air. The resulting synthetic orange-yellow g-C_3_N_4_ was then ground into a fine powder using a mortar and collected for use without further treatment [32, 33].

g-C_3_N_4_-P_25_ photocatalysts were synthesized as follows, g-C_3_N_4_ and P_25_, in a C:P = 1.5 (w/w) ratio, were dispersed in absolute ethyl alcohol, vigorously stirred for 4 h at 25 °C, then sonicated for 60 min at room temperature. After that, the product was collected and dried in a vacuum oven at 70 °C for 24 h, then cooled to room temperature and ground into a fine powder using a mortar for further use [34].

### Immobilization of g-C_3_N_4_-P_25_ or photosynthetic bacteria in calcium alginate (CA) beads

g-C_3_N_4_-P_25_-impregnated beads were prepared by adding 1 g of g-C_3_N_4_-P_25_ to 50 mL portions of 2% (w/v) sodium alginate (prepared by dissolving 1 g of sodium alginate in 50 mL distilled water at 35 °C). The resulting suspension was sonicated for 25 min at room temperature in a ultrasonic cleaning machine (Q5200DE) with 100HZ settings to disperse the catalyst uniformly in the sodium alginate solution. A 50 mL portion of the resulting mixture was injected dropwise into 150 mL of 2% CaCl2 solution using a 2.5 mL syringe to form calcium alginate-photocatalyst (CA+PC) beads, which were left in the 2% CaCl_2_ solution for 24h at 4℃ to harden. Calcium alginate-photosynthetic bacteria (CA+B) beads were formed in same manner, except the photocatalyst were replaced to photosynthetic bacteria to the alginate solution, and the mixing was performed by stirring rather than sonication [35–37].

### Preparation of the g-C_3_N_4_-P_25_/photosynthetic bacteria composite

g-C_3_N_4_-P_25_ (1 g) was mixed with a 4% (w/v) solution of sodium alginate (formed by dissolving 1 g sodium alginate in 25 mL distilled water at 35 °C), and the resulting suspension was sonicated as described above to disperse the catalyst uniformly. Photosynthetic bacteria (3g wet weight) and 25 mL distilled water were then added, and the resulting suspension was stirred to homogeneity. Calcium alginate beads containing the photocatalyst and bacteria (CA+B+PC beads) were then formed by dropping the suspension into CaCl_2_ solution, as also described above.

### Characterization of the g-C_3_N_4_-P_25_ and beads

XRD was performed on a Rigaku, Miniflex600 X-ray diffractometer, using a Cu Kα radiation (0.15406 nm) [38]. The diffraction patterns were recorded from 2θ = 10 to 80°. Brunner−Emmet−Teller (BET) surface area and porosity measurements were performed by N_2_ sorption-desorption analysis using an ASAP2020 instrument after degassing at 120 °C in vacuum [39]. Surface morphologies of encapsulated catalysts and photosynthetic bacteria in the alginate matrix were examined by SEM and energy dispersive X-ray analysis (EDAX) [36].

### Batch experiments for g-C_3_N_4_-P_25_ hybrid photocatalyst and CA+PC photocatalytic degradation azo-dye

Degradation experiments in 100mL serum bottle were conducted using 0.1g g-C_3_N_4_-P_25_ hybrid photocatalyst or 15g wet weight CA+PC (fresh) and 100mL 20mg/L or 50 mg/L reactive brilliant red X-3b solution. Each serum bottle was placed on magnetic stirrer mixed with a 2 cm stir bar under irradiation with 300w fluorescent lamp.

### Batch tests of the g-C_3_N_4_-P_25_/photosynthetic bacteria composite’s ability to degrade azo dyes and remove COD simultaneously

The ability of CA+B, CA+PC and CA+PC+B beads to degrade azo dyes and remove COD was tested by incubating 15 g (wet weight) samples of the beads in 100 mL portions of a solution containing 50 mg/L reactive brilliant red X-3b and 1500 mg/L glucose plus NH_4_Cl, NaHCO_3_ and trace elements at the concentrations used to cultivate the bacteria. These preparations (in static 100 mL serum bottles) were illuminated by halogen lamps set at the required distance from the bioreactors to maintain the cultures at about 35 °C. The reaction was continued for 96 hours.

### Sampling and analytical methods

Samples (6 mL) of the test solutions described above were taken at selected intervals and immediately filtered by passage through 0.45 μm polyvinylidene fluoride membrane filters. Then their absorption was measured by spectrophotometry at the maximal absorption wavelength in the UV-visible spectrum. To monitor changes in COD, portions of samples were centrifuged at 10,000 rpm for 20 min using a TGL16-G centrifuge (Shanghai, China), then the supernatants were analyzed according to national standard method HJ/T 399–2007.

To characterize degradation products, 25 mL samples were subjected to GC/MS analysis using a system consisting of a 6890 GC equipped with column and 5973 MSD (Agilent, USA), after esterification with 10 mL ethanol at 50 °C for 4 h in the presence of concentrated sulfuric acid and extraction with dichloromethane. The GC temperature program consisted of 2 min at 40 °C, followed by an 8 °C min^−1^ increase to 150 °C, held for 3 min, then a further 2 °C min^−1^ rise to 230 °C, held for 3 min, and a final 5 min^−1^ rise to 280 °C, held for 5 min.

## Results and discussion

### Photodegradation of reactive brilliant red X-3b by g-C_3_N_4_-P_25_

**Fig 1:**
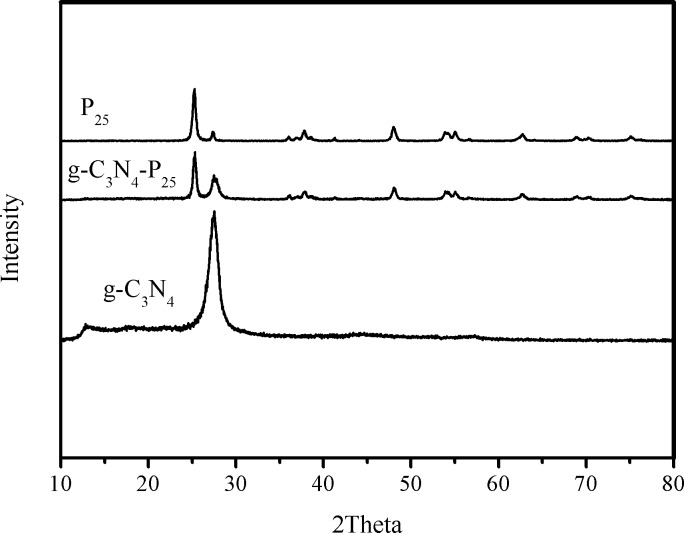
XRD data for hybrid photocatalyst.

The structure of g-C_3_N_4_-P_25_, P_25_ and g-C_3_N_4_ as determined by XRD analysis, is shown in Fig 1. All the typical XRD peaks of P_25_ and pure g-C_3_N_4_ are present in the spectrum of hybrid g-C_3_N_4_-P_25_. The strong peak at 27.5° represents stacking of conjugated aromatic systems, as occurs in the layered (002) crystal planes of graphitic materials. The weak peak at 12.9° is attributed to the (100) crystal plane, corresponding to the interlayer region. Peaks due to pure TiO_2_ are present at 25.3, 37.8, 48.0,53.9, 55.1, and 62.7°, corresponding to the (101), (004), (200), (105), (211) and (204) crystal planes of anatase TiO_2_, respectively [32, 38].

**Fig 2:**
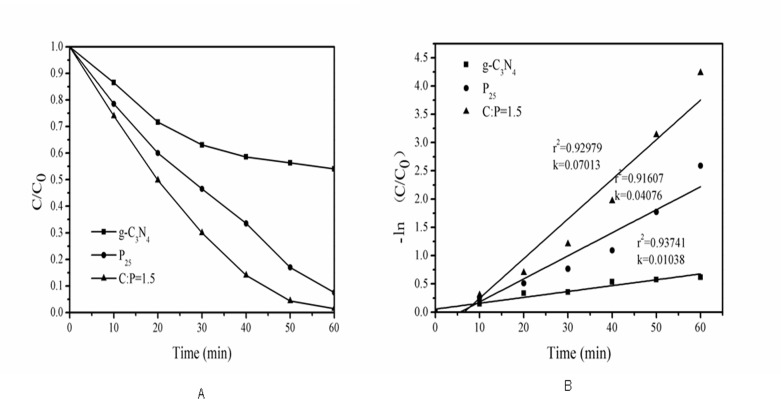
Study on photocatalytic performance. (A) Photodegradation of reactive brilliant red X-3b. (B) First-order kinetic modelling of its degradation.

The photocatalytic properties of g-C_3_N_4_-P_25_ were evaluated by monitoring the degradation of the azo dye reactive brilliant red X-3b and comparing the rates of degradation achieved with P_25_ and pure g-C_3_N_4_ under a halogen lamp. The experiments were performed without irradiation for 1 h to ensure that adsorption-desorption equilibrium had been reached. The adsorption rates of reactive brilliant red X-3b on g-C_3_N_4_-P_25_, P_25_ and pure g-C_3_N_4_ were 7.1, 9.6 and 3.5%, respectively. Fig 2A shows the photocatalysts’ activity in the degradation of reactive brilliant red X-3b under visible light irradiation. The hybrid g-C_3_N_4_-P_25_ exhibited a higher removal efficiency than either P_25_ or pure g-C_3_N_4_ alone, suggesting that the two catalyst systems are complementary. The rate constants of the catalytic processes were estimated by assuming that they exhibit first order kinetics and fitting the experimental data using the equation below [38]:

$$\text{ln}({\text{C}}_{\text{t}}/{\text{C}}_{0})=-\text{kt}$$ (1)

Here, C_t_ is the concentration at time t, C_0_ is the initial concentration, and k is the rate constant to be determined. As shown in Fig 2B, the hybrid g-C_3_N_4_-P_25_ had the highest rate constant of the tested photocatalysts.

**Fig 3:**
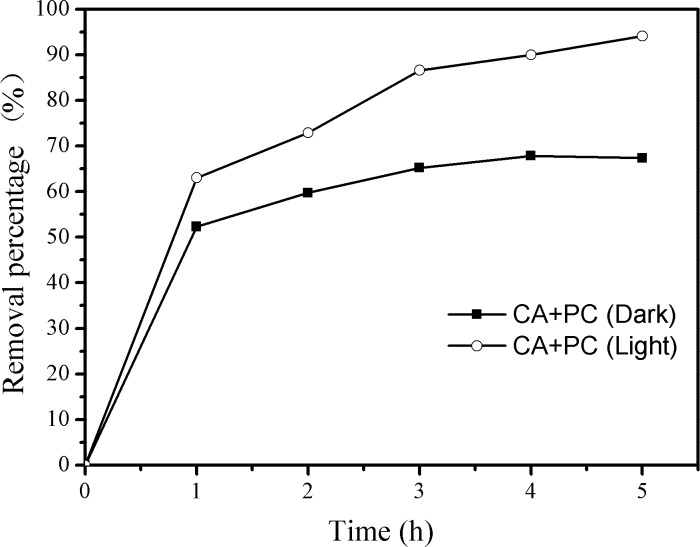
Removal of reactive brilliant red X-3 b by the immobilized photocatalyst in darkness (Dark) and under visible light illumination (Light).

The use of immobilized photocatalysts can facilitate otherwise cumbersome catalyst separation and recycling processes after wastewater treatment [36,40]. We therefore prepared immobilized CA+PC catalysts in which g-C_3_N_4_-P_25_ is incorporated into calcium alginate beads. The resulting immobilized photocatalyst exhibited good levels of dye adsorption and photocatalytic degradation. Its capacity to remove dye by adsorption was investigated by performing control experiments in darkness, and its combined adsorptive and photocatalytic removal efficiency was investigated by performing experiments under visible light irradiation. As shown in Fig 3, the efficiency of dye removal was around 67% in darkness, but that under illumination was around 94% after 5h. During the first hour of the experiments with illumination, photocatalytic degradation was less important in dye removal than adsorption, which was facilitated by the carboxyl and hydroxyl groups on the surfaces of the calcium alginate beads and the high specific surface area of the g-C_3_N_4_-P_25_ hybrid.

### SEM and BET analysis

**Fig 4:**
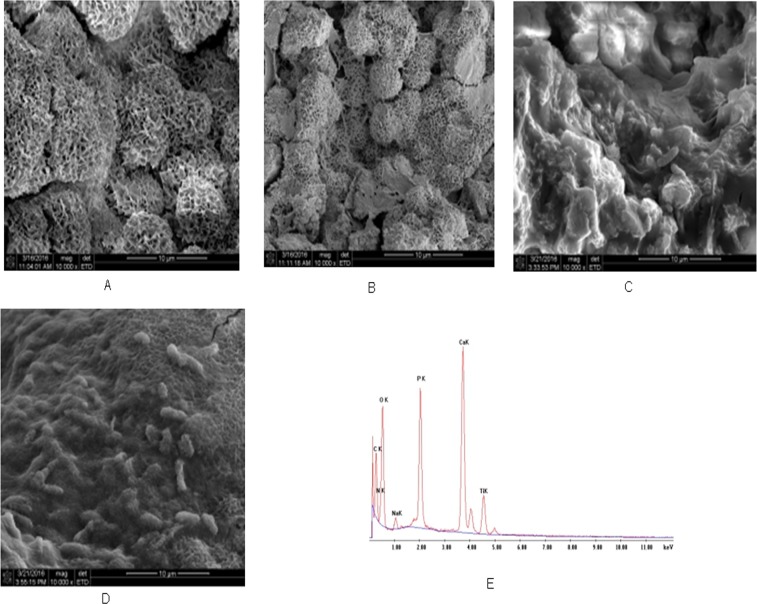
Characterization of the materials. SEM images of CA (A), CA+PC (B), CA+B (C), and CA+B+PC (D) beads, and EDXA spectra of CA+B+PC (E).

The CA, CA+PC, CA+B, CA+B+PC beads were examined by SEM to characterize the carriers’ surface morphology, to detect the presence of nanoparticles and photosynthetic bacteria, and to determine the distribution of both in the polymeric matrix [36]. As shown in Fig 4A, there were channels on the surfaces of the CA beads, which is beneficial for microbial metabolism. Representative SEM images of CA+PC (Fig 4B) and CA+B (Fig 4C) showed that the particles and photosynthetic bacteria were generally quite evenly distributed in the carriers. Moreover, as shown in Fig 4D, the content of photosynthetic bacteria in the polymeric matrix was relatively high. EDXA spectroscopy was used to perform a quantitative compositional analysis of the CA+B+PC beads (Fig 4E), confirming the presence of g-C_3_N_4_-P_25_. Ideally, most of the photosynthetic bacteria would be encapsulated in the sodium alginate gel to protect them from strongly oxidizing radicals. The photocatalyst was uniformly dispersed within the carriers, but presumably only material on the exterior of the beads would contribute significantly to the degradation of recalcitrant or non-biodegradable organic matter by releasing free radicals under irradiation. These results are consistent with the hypothesis that the CA+B+PC beads can simultaneously degrade azo dyes and reduce the COD of wastewater by combining radical oxidation and microbial metabolism.

**Fig 5:**
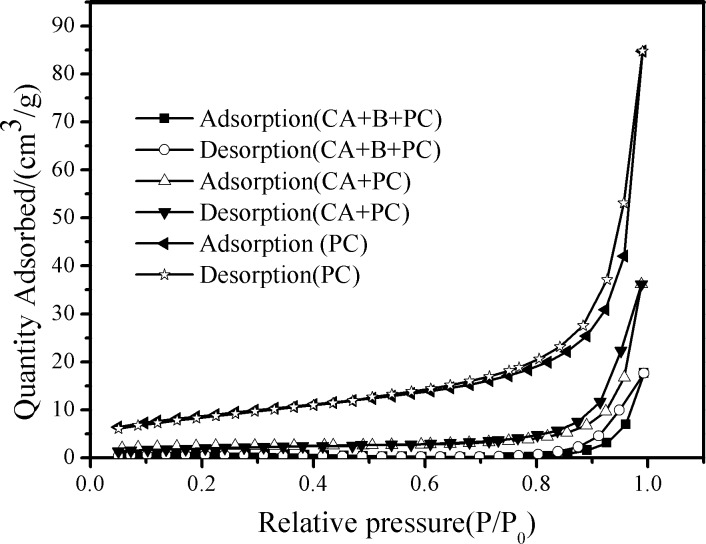
N_2_ sorption isotherms for CA+B+PC, CA+PC and PC beads.

As shown in Fig 5, all of the samples exhibited type IV adsorption-desorption isotherms according to IUPAC classification, indicating the presence of mesopores. A type H_3_ hysteresis loop is observed at high P/P_0_ values of 0.8–1.0, indicating the presence of slit-shaped pores resulting from the aggregation of plate-like particles [32]. Table 1 shows the pore volume and pore size distribution of PC, CA+B CA+PC, CA+B+PC, which were determined on the basis of N_2_ adsorption-desorption isotherms using the BJH (Barrett-Joyner-Halenda) method [39]. The specific surface area of CA+PC was higher than that of CA+B+PC because of the bacterial coverage of the latter beads. The specific surface area of CA+B was so low that all the porous sites of the calcium alginate beads were occupied by photosynthetic bacteria. Noteworthyly,the pore size displayed in SEM images and calculated values from N_2_ adsorption-desorption isotherms (as given in Table 1) are very different.This phenomenon can be explained from the following point: The SEM image shows the pore distribution on the surface of the beads. In the BET analysis, the beads was ground into a powder and the pore structure of the carrier was destroyed. The mesopores measured by BET analysis were probably due to the presence of g-C_3_N_4_-P_25_. Combining SEM and BET analysis, it is possible to predict the presence of both micropores and mesopores in the composite.

**Table 1:** Textural properties of the CA+B+PC, CA+PC, CA+B and PC beads.

Characteristics	CA+B+PC	CA+PC	CA+B	PC
**Surface area (m**^**2**^**/g)**	2.3856	7.7553	0.0212	31.2556
**Average pore diameter (nm)**	45.73329	28.84185		16.77893
**Pore volume (cm**^**3**^**/g)**	0.027276	0.055920		0.131109

### Simultaneous decolorization and COD reduction by the g-C_3_N_4_-P_25_/photosynthetic bacteria composite

**Fig 6:**
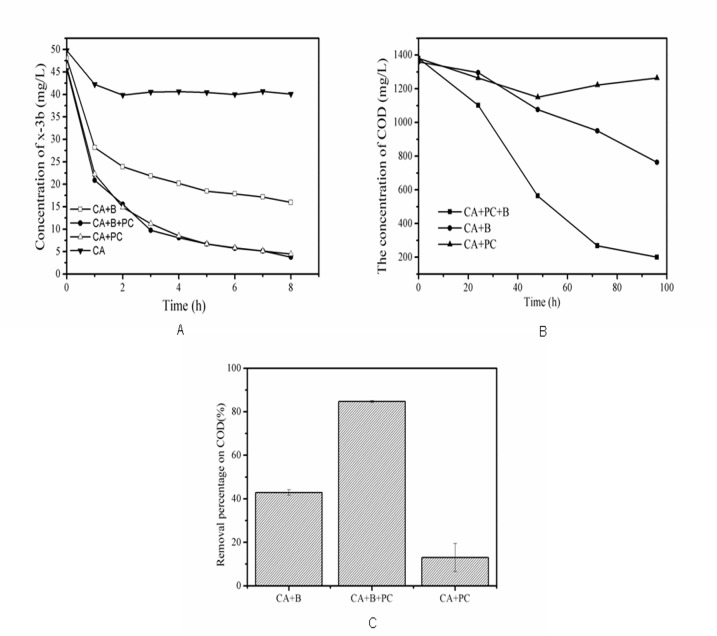
Simultaneous decolorization and COD reduction by materials. (A)Residual concentrations of reactive brilliant red X-3b. (B) Measured COD at different time points in reactions performed with CA+B, CA+B+PC, CA+PC. (C)COD level (as a percentage of the initial value) after 96h in each case (n = 3).

As shown in Fig 6A, when dye-contaminated synthetic wastewater with a high COD was treated with the composite material, rapid decolorization occurred within 60 minutes; this was largely attributed to the material’s adsorptive properties. As the reaction time increased, the rate of decolorization fell. After 8 hours, the efficiencies of dye removal using CA alone, CA+B, CA+PC, CA+B+PC were 20%, 68%, 92% and 94%, respectively. The efficiency of biological decolorization before 8h was very slow, possibly because the photosynthetic bacteria required some time to adapt to the new environment. However, near-complete dye removal was achieved over 24 hours in the experiments using CA+B, demonstrating that efficient removal of reactive brilliant red X-3b can be achieved by biosorption and biodegradation. The photocatalytic degradation of the azo dye proceeded with first-order kinetics; rate constants of 0.29216 and 0.30178 h^-1^ were determined for decolorization by CA+PC and CA+B+PC, respectively. These two values are very similar, suggesting that the rate of the photocatalytic process was substantially greater than that of biodegradation. Therefore, photocatalysis was the primary means of dye degradation during the first 8 h. However, the residual COD after 96 hours was around 200 mg/L when using the CA+B+PC beads, whereas that achieved with the CA+PC beads was 1264mg/L (Fig 6B). COD degradation experiments of CA+B+PC, CA+B and CA+PC were done 5 times in parallel, corresponding to COD reductions of 84.7%, 43.8% and 13%, respectively (Fig 6C). This difference can be attributed to the contribution of the photosynthetic bacteria. Photocatalysis alone cannot completely mineralize organic matter because the formation of free radicals is inhibited partly by the presence of bicarbonate and other anions [13]. Consequently, partially degraded intermediates accumulated in the experiments performed with the CA+PC beads, and the COD was only reduced by a small amount. Conversely, when photocatalysis is combined with microbial metabolism, the initial degradation of the dyes yields products that are rapidly taken up by the microorganisms inside the carrier and immediately mineralized [21]. Additionally, glucose was responsible for a significant fraction of the wastewater’s overall COD; because this sugar is readily taken up by living cells, the encapsulated bacteria rapidly mineralized it and removed its contribution. This is demonstrated by the fact that the CA+B beads, which have no photocatalyst, achieved an appreciable COD reduction, yielding a residual COD of around 764 mg/L (Fig 6B)—substantially higher than that achieved with the CA+B+PC beads but much lower than that for the CA+PC beads. Overall, these results indicate that combining photocatalysis with microbial metabolism enables efficient COD reduction and dye removal, and that the photocatalytic generation of oxidative radicals had little adverse effect on the microorganisms, presumably because they were protected by encapsulation in the carrier. In order to ensure the activity of microorganisms, The secondary degradation of the composites was studied. It was found that the second decolorization rate is the first 99.1%, COD removal percentage accounted for the first 98%, indicating bacteria activity were not affected.

### UV-visible absorption spectra and GC-MS analysis of intermediates

**Fig 7:**
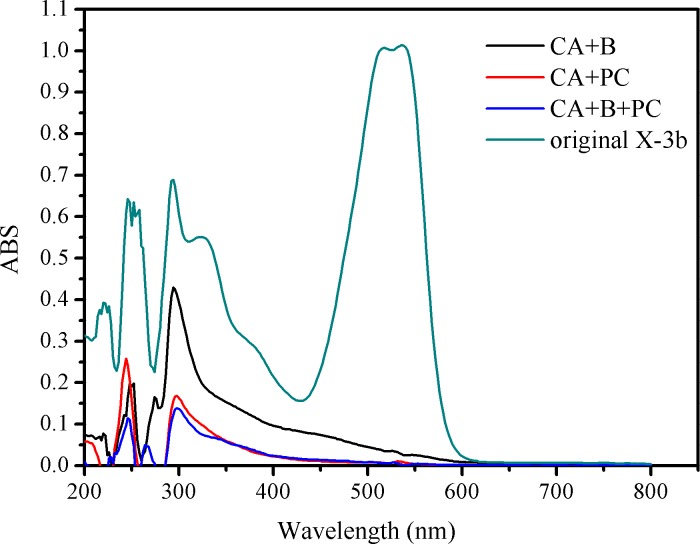
Changes in the UV-visible absorption spectra of brilliant red X-3b.

**Fig 8:**
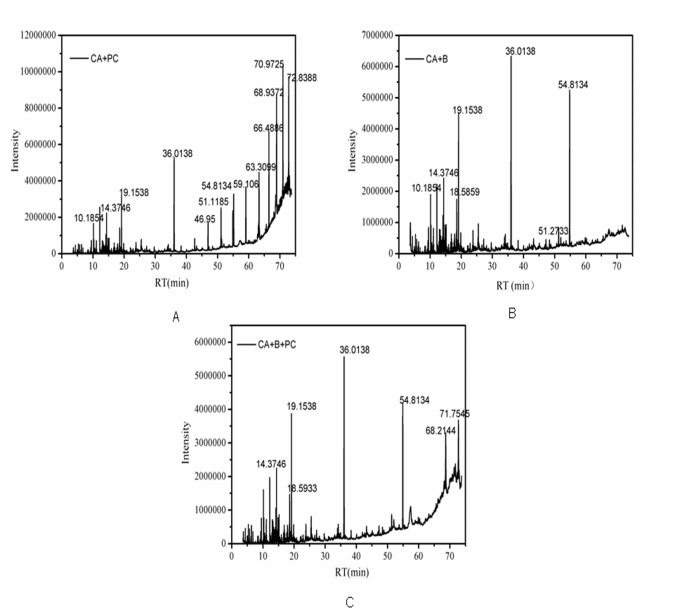
The GC spectra of a solution containing 100mg/l reactive brilliant red X-3b after 84 hours’ incubation. (A) CA+B. (B) CA+PC. (C) CA+B+PC.

**Fig 9:**
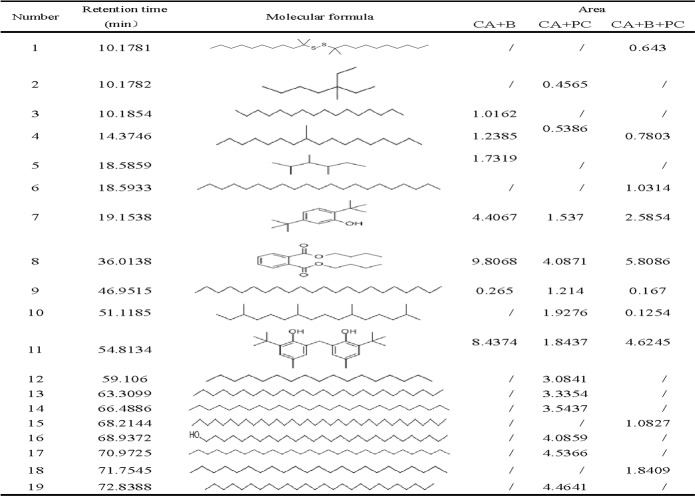
Intermediates in the degradation of reactive brilliant red X-3b as characterized by GC-MS.

Although the strong reductions in the residual concentrations of reactive brilliant red X-3b and COD achieved with the new composite strongly demonstrate its degradation ability, the mechanistic details of the degradation process remain unclear. To shed some light on these details, UV-visible absorption spectroscopy and GC-MS were used to investigate the intermediates present in the reaction mixtures formed after 84 hours’ treatment of synthetic wastewater samples with CA+B, CA+PC, and CA+B+PC. Fig 7 shows the UV-Vis spectrum of the synthetic wastewater before treatment; it features a prominent absorption peak at 540 nm due to the conjugated chromophore of reactive brilliant red X-3b, and additional peaks corresponding to benzene and naphthalene rings in the ultraviolet region at 245 nm, 283 nm and 324 nm. After reaction for 84 h, the peaks at 540 nm had disappeared, indicating that the conjugated system (i.e. the azo dye motif) had been destroyed. Furthermore, the peaks at 324nm had disappeared, indicating that the naphthalene rings had been degraded. The peaks due to benzene rings were less intense than in the spectrum of untreated wastewater in all cases, but the reduction in their intensity in the CA+B system was less pronounced than in the CA+PC and CA+B+PC systems, indicating that partially degraded intermediates featuring benzene rings were not readily metabolized by the photosynthetic bacteria. Figs 8 and 9 indicate that aromatic ring compounds were the main oxidation products formed when using CA+B, while CA+PC primarily formed long-chain alkanes. It is assumed that the first step in the combined photocatalytic and biocatalytic degradation of X-3b catalyzed by CA+B+PC is the conversion of the azo dye into aniline and phenol derivatives. The aromatic rings of these products are then presumably attacked by free radicals generated by the photocatalyst, leading to the formation of linear alkyl compounds, thereby preventing aromatic hydrocarbon inhibition of bacterial metabolism. The alkyl products could then be degraded by the photosynthetic bacteria.

## Conclusion

A novel visible-light-responsive composite photocatalyst incorporating both the photoactive material g-C_3_N_4_-P_25_ and photosynthetic bacteria was successfully prepared by self-assembly. Visible light excites the g-C_3_N_4_-P_25_ photocatalyst on the beads’ surfaces, resulting in electron transfer from the valence band to the conduction band and the generation of electron–hole pairs. This in turn generates free radicals, which perform the initial oxidative degradation of the azo dyes. The initial degradation products, together with high-COD compounds such as glucose can freely enter the beads’ internal spaces, where they are degraded by further radical oxidation and microbial metabolism. The composite catalyst system efficiently decolorized the synthetic wastewater and reduced its COD, demonstrating that advantages of the CA+B+PC beads and the combination of photocatalysis with biodegradation.

## Supporting information

S1 File Raw data. (XLS)

## Abbreviations

COD: Chemical Oxygen Demand
AOPs: Advanced oxidation processes
XRD: X-ray diffraction
SEM: Scanning electron microscopy
GC-MS: Gas chromatography-mass spectrometry
CA: Calcium alginate
CA+PC: Calcium alginate-photocatalyst
CA+B: Calcium alginate-photosynthetic bacteria
CA+B+PC: Calcium alginate beads containing the photocatalyst and bacteria
BET: Brunner−Emmet−Teller
EDAX: Energy dispersive X-ray analysis
BJH: Barrett-Joyner-Halenda
